# Polish translation and validation of the Pelvic Organ Prolapse/Urinary Incontinence Sexual Questionnaire, IUGA-Revised (PISQ-IR)

**DOI:** 10.1007/s00192-017-3539-5

**Published:** 2017-12-29

**Authors:** Magdalena Emilia Grzybowska, Justyna Piaskowska-Cala, Dariusz Grzegorz Wydra

**Affiliations:** Department of Gynecology, Gynecologic Oncology and Gynecologic Endocrinology, Medical University of Gdańsk, Kliniczna 1a, 80-402 Gdańsk, Poland

**Keywords:** Female sexual function, Pelvic organ prolapse, Pelvic Organ Prolapse/Urinary Incontinence Sexual Questionnaire (PISQ), Quality of life, Urinary incontinence

## Abstract

**Introduction and hypothesis:**

The aim of the study was to translate into Polish the Pelvic Organ Prolapse/Incontinence Sexual Questionnaire, IUGA-Revised (PISQ-IR), which evaluates sexual function in sexually active (SA) and not SA (NSA) women with pelvic floor disorders (PFD), and to validate the Polish version.

**Methods:**

After translation, back-translation and cognitive interviews, the final version of PISQ-IR was established. The study group included 252 women with PFD (124 NSA and 128 SA). All women underwent clinical evaluation and completed the PISQ-IR. For test–retest reliability, the questionnaire was administered to 99 patients twice at an interval of 2 weeks. The analysis of criterion validity required the subjects to complete self-reported measures. Internal consistency and criterion validity were assessed separately for NSA and SA women for the PISQ-IR subscales.

**Results:**

The mean age of the women was 60.9 ± 10.6 years and their mean BMI was 27.9 ± 4.9 kg/m^2^. Postmenopausal women constituted 82.5% of the study group. Urinary incontinence (UI) was diagnosed in 60 women (23.8%), pelvic organ prolapse (POP) in 90 (35.7%), and UI and POP in 102 (40.5%). Fecal incontinence was reported by 45 women (17.9%). The PISQ-IR Polish version proved to have good internal consistency in NSA women (*α* 0.651 to 0.857) and SA women (*α* 0.605 to 0.887), and strong reliability in all subscales (Pearson’s coefficient 0.759–0.899; *p* < 0.001). Criterion validity confirmed moderate to strong correlations between PISQ-IR scores and self-reported measures in SA subscales, as well the SA summary score, and weak to moderate correlations in NSA women.

**Conclusions:**

The PISQ-IR Polish version is a valid tool for evaluating sexual function in women with PFD.

**Electronic supplementary material:**

The online version of this article (10.1007/s00192-017-3539-5) contains supplementary material, which is available to authorized users.

## Introduction

A considerable number of authors have focused their attention on the quality of sexual life of patients with pelvic floor disorders (PFD). There has been an increase in the number of studies on sexual function in patients with PFD after both, surgical interventions and conservative management. In recent years the popularity of the Pelvic Organ Prolapse/Incontinence Sexual Questionnaire (PISQ) has grown. Originally introduced in 2001 [1], PISQ remains the only condition-specific tool to assess sexual function in women with urinary incontinence (UI) and/or pelvic organ prolapse (POP). PISQ has been classified as ‘grade B’ according to the three-grade scale of recommended questionnaires for the evaluation of symptoms and impact of POP on health-related quality of life (QoL) [2].

During the International Urogynecological Association (IUGA) Female Sexual Dysfunction Roundtable in 2008, suitable modifications were made, giving rise to the PISQ IUGA-revised (PISQ-IR) questionnaire. The new version of the questionnaire can also be administered to sexually inactive women, allowing analysis of the causes of sexual abstinence, including UI, POP, fecal incontinence (FI), other health problems, and lack of partner or interest in sexual activity [3].

The success of a therapeutic process is no longer determined only on the basis of its anatomical effect, but also in terms of its effects on the functional roles of the vagina and the genital organs. Questionnaires are the main tools for QoL evaluation, including sexual function, as they allow objective analysis and comparison of the results obtained [4–6]. In order for a new language version of any questionnaire to be used, both compliance with the original and the capacity to analyze a given phenomenon in another population need to be confirmed. Accurate translation does not always allow the questionnaire to be used or a specific meaning to be conveyed. Thus, a process of translation and validation is required to adjust the translation to the linguistic and cultural differences and nuances, while remaining faithful to the original.

In our center, we validated the original version of PISQ [7], and that experience proved to be invaluable for future reference. Bearing in mind that sexual function is a vital aspect of the therapeutic effect in PFD patients, we chose to translate and validate the latest questionnaire for women with PFD, i.e. PISQ-IR.

## Materials and methods

### Validation of the questionnaire

The IUGA Translation Protocol for PISQ-IR was followed during the validation process. The local Ethics Committee approved the study and the Ethics Review Board approval was reported to IUGA.

The initial translation of the item pool was performed by two translators (M.E.G. and an independent translator), followed by community reviews in cognitive interviews conducted by J.P.C., who did not translate the instrument. Ten cognitive interviews were conducted during one-on-one sessions. Back translation was performed by an independent translator. The final translation and the back translation into English were submitted to the IUGA Translation Working Group for review.

The study included women presenting with complaints of UI and/or POP and/or FI and referred to a university-based gynecological clinic. The inclusion criteria were as follows: age >18 years, not pregnant, and able to read/write and understand Polish. The exclusion criteria were: vulvodynia, painful bladder syndrome, and chronic pelvic pain. Both sexually active (SA) and not SA (NSA) women were recruited. Eligible women gave their informed consent and completed the PISQ-IR. For analysis of criterion validity the women were required to complete other related self-reported measures: the Incontinence Severity Index (ISI), the short form of the Pelvic Floor Distress Inventory (PFDI-20), a single prolapse question (#35) from the Epidemiology of Prolapse and Incontinence Questionnaire (EPIQ), and the Female Sexual Function Index (FSFI). Outside the protocol, the women were required to complete the King’s Health Questionnaire (KHQ) and the 36-item Short Form Health Survey (SF-36). KHQ was analyzed only in patients with UI. Medical history on treatment and surgical interventions was obtained. Patient clinical and physical characteristics were collected according to the IUGA protocol. Pelvic floor examination included assessment of POP using the POP-Q system, assessment of the ability to contract the pelvic floor using the Oxford Grading Scale (*0* no contraction, *1* flicker, *2* weak, *3* moderate, *4* good, *5* strong contraction), and assessment of the pelvic floor muscles (PFM) according to the International Continence Society recommendations (normal, overactive, underactive, non-functioning) [8].

For reliability assessment, the questionnaire was administered twice, at an interval of 2 weeks. PISQ-IR was mailed 2 weeks before hospital admission for the first administration, and the women completed the questionnaire during their stay at the clinic for the second administration. Internal consistency, test–retest reliability, and criterion-related validity were assessed in PISQ-IR subscales, separately for NSA and SA women. The outcome measures were the scores of the particular scales and subscales of the applied questionnaires and patient characteristics.

### Applied questionnaires

In the first core branching item of PISQ-IR, the woman provides subjective assessment of whether or not she considers herself SA. PISQ-IR consists of two parts. Part 1, for NSA women, includes 12 items in the following scales: Condition Specific (NSA-CS, 3 items), Partner-related (NSA-PR, 2 items), Global Quality (NSA-GQ, 4 items), and Condition Impact (NSA-CI, 3 items). Higher scores indicate greater impact of the condition on sexual inactivity. Question #2 allows the woman to identify her reason(s) for sexual inactivity, e.g. bladder, bowel or prolapse problems. Part 2, for SA women, includes 21 items in the following scales: Arousal, Orgasm (SA-AO, 4 items), Partner-related (SA-PR, 3 items), Condition Specific (SA-CS, 3 items), Global Quality (SA-GQ, 4 items), Condition Impact (SA-CI, 4 items), and Desire (SA-D, 3 items). Higher scores indicate better sexual function. An Excel file was provided by IUGA to assist with data entry, storage and scoring. Mean calculation scores of PISQ-IR and a transformed sum score to a 0–100 range were used. A single summary score for the SA group was calculated.

FSFI is a self-administered, 19-item generic questionnaire, characterizing six domains of sexual function: desire, arousal, lubrication, orgasm, satisfaction, pain and total score. Higher scores indicate better sexual function [9, 10]. PFDI-20 consists of three scales: the Urinary Distress Inventory (UDI), the Pelvic Organ Prolapse Distress Inventory (POPDI), and the Colorectal-Anal Distress Inventory (CRADI). The score for each scale ranges from 0 (the smallest impact) to 100 (greatest distress) with a total range of 0–300 [11]. Both FSFI [12] and PFDI-20 have been validated in Polish. The ISI consists of two questions, one on how often the respondent experiences urine leakage and the other on how much urine she loses each time. The three-level Sandvik severity index, that categorizes UI into slight, moderate and severe, was used [13]. In EPIQ question #35, the respondent reports if she has a sensation of bulge in the vagina or something falling out of the vagina, and how much bother it causes on a scale from 0 to 10 (0 not at all, 10 greatly) [14].

KHQ is a condition-specific questionnaire to assess QoL in patients with UI. It includes 21 questions in eight domains: General health, Incontinence impact, Role limitations, Physical limitations, Social limitations, Personal relationships, Emotions, and Sleep/Energy. The scores for each domain range from 0 to 100, with 0 representing the best and 100 the worst possible health status [15]. The Polish version of KHQ (information, contact and consent) is available from MAPI Research Trust, Lyon, France (e-mail PROinformation@mapi-trust.org; www.mapi-trust.org). SF-36 is a 36-item, patient-reported survey of patient health status. It consists of eight scaled scores, which are the weighted sums of the questions in each section. Lower scores correspond to more disability, with 0 ‘maximum disability’ and 100 ‘no disability’. The eight sections include physical functioning, role-physical, bodily pain, general health, vitality, social functioning, role-emotional, mental health and additionally reported health transition. A license to use the questionnaire was obtained from OptumInsight Life Sciences, Inc.

### Statistical analysis

Statistical analysis was performed using IBM SPSS Statistics 24.0 (IBM Corp., Armonk, NY). Continuous variables are expressed as means ± standard deviations, categorical variables as percentages of the total group. A *p* value of <0.05 was considered statistically significant. Student’s *t* test was used for quantitative variables, and Pearson’s chi-squared test and Fisher’s exact test (if the expected value in any of the cells was <5) for qualitative variables. Correlations between PISQ-IR subscale scores and the scores of other questionnaires and clinical measures were calculated using Spearman’s rank correlation because many variables had non-normal distributions. A rho of <0.3 was classified as ‘weak’, 0.3–0.5 as ‘moderate’, and >0.5 as ‘strong’. Cronbach’s *α* was used to measure reliability of each PISQ-IR subscale. Pearson’s correlation coefficient (*r*) was used to measure test–retest reliability.

The number of women recruited into the study was based on the rule of thumb in psychometric work to the effect that maybe at least 10 subjects^2^ for each variable included in the analysis. Therefore, the study required the enrollment of a total of 220 women: 120 SA and 100 NSA.

## Results

A total of 307 patients were approached but due to a lack of clinical data and unreturned surveys, 252 (128 SA and 124 NSA) women were enrolled in the study. The response rate was 82.1%. The women were diagnosed as follows: 90 (35.7%) with POP, 60 (23.8%) with UI, and 102 (40.5%) with both POP and UI. FI was reported by 45 of the women. The mean patient age was 60.9 ± 10.6 years and their mean body mass index (BMI) was 27.9 ± 4.9 kg/m^2^. Of the 252 women, 208 (82.5%) were postmenopausal. In the study group, the majority of women – 213 (84.5%) were deemed to be eligible for subsequent surgical management, and 39 (15.5%) for conservative management that included pessaries – 10 (4.0%), PFM training – 8 (3.2%), and overactive bladder syndrome therapy – 21 (8.3%). The characteristics of the women enrolled in the study are presented in Table 1. Respondents did not differ from non-respondents in terms of BMI and education, but the non-respondents were significantly older (64.5 ± 10.0 vs. 60.9 ± 10.6, *p* = 0.02).

**Table 1:** Characteristics of the women enrolled in the study

	Sexually active (*n* = 128)	Not sexually active (*n* = 124)	*p* value
Age (years)	56.3 ± 10.8	65.7 ± 7.9	<0.001^b^
BMI (kg/m^2^)	27.4 ± 5.0	28.4 ± 4.7	0.10^b^
Education			
Primary	6 (4.7)	13 (10.5)	0.80^c^
Secondary	76 (59.4)	79 (63.7)	
Higher	46 (35.9)	32 (25.8)	
Parity	2.3 ± 0.9	2.3 ± 1.5	0.94^b^
Delivery mode^a^			
Vaginal	113 (88.3)	118 (95.9)	0.06^d^
Cesarean section	3 (2.3)	1 (0.8)	
Both	12 (9.4)	4 (3.3)	
Postmenopausal	92 (71.9)	116 (93.5)	<0.001^c^
POP-Q stage			
0	4 (3.1)	4 (3.2)	0.04^d^
I	5 (3.9)	12 (9.7)	
II	40 (31.3)	25 (20.2)	
III	69 (53.9)	63 (50.8)	
IV	10 (7.8)	20 (16.1)	
Clinical diagnosis			
POP	45 (35.2)	45 (36.3)	0.98^c^
UI	31 (24.2)	29 (23.4)	
UI and POP	52 (40.6)	50 (40.3)	
Fecal incontinence	15 (11.7)	30 (24.2)	0.01^c^
Prior gynecological surgery			
None	104 (81.2)	100 (80.6)	0.78^c^
Hysterectomy	23 (18.0)	24 (19.4)	0.78^c^
Prolapse surgery	24 (18.7)	24 (19.4)	0.90^c^
UI surgery	9 (7.0)	15 (12.1)	0.17^c^
Oxford scale grade			
No contraction/flicker	57 (44.5)	68 (54.8)	0.19^c^
Weak contraction	26 (20.3)	25 (20.2)	
Moderate contraction	25 (19.5)	13 (10.5)	
Good/strong contraction	20 (15.6)	18 (14.5)	
Pelvic floor muscles			
Normal	92 (71.9)	71 (57.3)	0.06^d^
Overactive	1 (0.8)	3 (2.4)	
Underactive	13 (10.1)	23 (18.5)	
Non-functioning	22 (17.2)	27 (21.8)	

The data presented are number (%) of women or mean ± SD

^a^One woman in the NSA group was nulliparous

^b^Student’s *t* test

^c^Pearson's chi-squared test

^d^Fisher's exact test

### Assessment of internal consistency

The PISQ-IR Polish version proved to have good internal consistency with Cronbach’s *α* ranging from 0.651 to 0.857 in NSA women and from 0.605 to 0.887 in SA women (Table 2). Strong internal consistency was observed for two subscales in NSA women (Global Quality and Condition Impact), and in five of six subscales in SA women (Arousal Orgasm, Condition Specific, Global Quality, Condition Impact, Desire). Internal consistency was moderate in SA women in the Partner-Related scale and in NSA women in the Condition-Specific scale. Excellent internal consistency was confirmed for the summary score among SA women.

**Table 2:** Calculated scores and Cronbach’s *α* for each subscale

Scale	No. of items	Calculated score (mean ± SD)	*α*	95% Confidence interval	
				Upper bound	Lower bound
Not sexually active					
Partner-related	2	2.80 ± 0.98	n.a.	–	–
Condition Specific	3	2.00 ± 0.90	0.651	0.53	0.75
Global Quality	4	2.57 ± 1.19	0.857	0.81	0.89
Condition Impact	3	2.21 ± 1.09	0.848	0.80	0.89
Sexually active					
Arousal, Orgasm	4	3.06 ± 0.80	0.745	0.67	0.81
Partner-related	3	3.30 ± 0.47	0.605	0.47	0.71
Condition Specific	3	4.15 ± 0.93	0.714	0.62	0.79
Global Quality	4	3.06 ± 1.08	0.887	0.85	0.92
Condition Impact	4	2.69 ± 0.85	0.847	0.80	0.89
Desire	3	2.79 ± 0.71	0.726	0.64	0.80
Summary score	21	3.03 ± 0.57	0.900	0.87	0.93

*n.a.* not assessed (only two items in the scale)

### Assessment of stability

Test–retest analysis was performed in 99 patients (43 NSA and 56 SA). The analysis revealed a strong correlation between the paired test–retest scores for all subscales in both NSA and SA women, with Pearson’s coefficients ranging from 0.759 to 0.899, *p* < 0.001 (Table 3).

**Table 3:** Pearson’s correlation coefficient for each PISQ-IR subscale in NSA and SA women

Scale	No. of women	Δ Transformed sum score (mean ± SD)	*r*
Not sexually active			
Partner-related	43	3.1 ± 23.9	0.759
Condition Specific	43	7.5 ± 23.4	0.779
Global Quality	43	−2.6 ± 19.4	0.835
Condition Impact	43	2.8 ± 16.3	0.899
Sexually active			
Arousal, Orgasm	56	1.9 ± 10.7	0.857
Partner-related	54	0.6 ± 10.7	0.813
Condition Specific	54	1.6 ± 15.5	0.812
Global Quality	56	4.1 ± 15.4	0.817
Condition Impact	56	3.9 ± 18.7	0.782
Desire	56	1.1 ± 9.4	0.865

*p* < 0.001 for all subscales

### Assessment of criterion-related validity

#### Sexually inactive women

Criterion-related validity evaluated with clinical measures revealed that pelvic floor tone and BMI were not correlated with PISQ-IR scores in NSA women. Weak statistically significant correlations between POP-Q stage and Oxford scale grade versus NSA-PR and NSA-GQ subscale scores were found. Analysis of the correlations with other self-administered questionnaires revealed positive correlations between PFDI-20 scores and NSA-CS and NSA-CI scores (rho = 0.351 and 0.309, respectively), and between EPIQ question #35 scores and NSA-GQ and NSA-CI scores (rho = 0.355 and 0.488, respectively). Higher PFD-related distress resulted in higher impact of the condition on sexual function. Furthermore, scores for selected domains of the SF-36 questionnaire showed negative correlations with the PISQ-IR subscale scores. Lower SF-36 scores (more disability) correlated with higher NSA subscale scores, indicating a greater impact of the disease on sexual inactivity. KHQ responses were analyzed only among UI patients. The results confirmed a positive correlation between selected KHQ domain scores and NSA subscale scores. Higher KHQ scores (worse health status) indicated a greater impact of the disease on sexual function in NSA women. NSA-GQ scores were correlated only with the scores of isolated domains from the questionnaires applied, i.e. SF-36 Social functioning, KHQ-Personal limitations and EPIQ question #35. Detailed results are presented in Table 4.

**Table 4:** Criterion validity: scale score correlations in NSA women

	Condition specific	Partner-related	Global quality	Condition impact
POP-Q stage	0.102	−0.187*	−0.184*	0.004
Pelvic floor tone	−0.080	0.136	−0.197*	−0.112
Oxford scale	0.068	−0.204*	0.242**	0.164
Incontinence Severity Index	0.088	0.056	0.034	0.123
PFDI-20	0.351**	−0.072	0.125	0.309**
EPIC question 35^a^	0.236	−0.186	0.355**	0.488**
Body mass index (kg/m^2^)	−0.032	0.155	0.012	−0.102
SF-36				
Physical functioning	−0.247**	−0.067	−0.020	−0.147
Role-physical	−0.271**	−0.017	−0.028	−0.172
Role-emotional	−0.130	−0.016	−0.048	−0.144
Vitality	−0.272**	0.037	−0.154	−0.243*
Mental health	−0.315**	−0.065	−0.190*	−0.362**
Social functioning	−0.198*	−0.073	−0.217*	−0.294**
Bodily pain	−0.169	−0.054	−0.152	−0.100
General health	−0.224*	−0.085	−0.047	−0.213*
KHQ^b^				
General health perception	0.170	0.006	−0.048	0.058
Incontinence impact	0.111	−0.011	−0.159	0.201
Role limitations	0.149	−0.111	−0.092	0.233*
Physical limitations	0.132	−0.117	−0.058	0.230*
Social limitations	0.106	0.029	0.001	0.173
Personal limitations	0.384*	−0.337	0.373*	0.602**
Emotional problems	0.362**	−0.153	0.163	0.500**
Sleep/energy	0.404**	0.133	−0.105	0.164
Symptom severity	0.220	0.054	−0.071	0.208

*POP* pelvic organ prolapse, *POP-Q* Pelvic Organ Prolapse Qualification, *PFDI-20* Pelvic Floor Distress Inventory, *EPIC* Epidemiology of Prolapse and Incontinence Questionnaire, *KHQ* King’s Health Questionnaire, *SF-36* 36-item Short Form Health Survey

*p < 0.05, **p < 0.01

^a^Analysis only in patients with POP

^b^Assessed only in patients with urinary incontinence

### Sexually active women

In the SA group, of the clinical measures, POP-Q stage, Oxford scale grade and BMI were not significantly correlated with the PISQ-IR results. Only pelvic floor tone showed a weak statistically significant negative correlation with SA-GQ scores (rho = −0.183), indicating that underactive or non-functioning PFM were associated with worse sexual function in SA-GQ subscale. The analysis also revealed a weak statistically significant negative correlation between ISI and SA-CS and SA-CI scores (rho = −0.361 and −0.225, respectively) and PFDI-20 scores with five of six subscales in SA women, i.e. SA-AO (rho = −0.333), SA-PR (rho = −0.343), SA-CS (rho = −0.284), SA-GQ (rho = −0.357) and SA-CI (rho = −0.367). Only the SA-D scores were not correlated with the PFDI-20 results. EPIQ question #35 scores were not significantly correlated with condition-specific subscale scores (SA-CS and SA-CI).

In the SA group, all FSFI domain scores and FSFI total score were positively correlated with SA-AO, SA-GQ, SA-CI and SA-D scores, whereas SA-PR scores were significantly correlated with FSFI total score (rho = 0.229) and FSFI Arousal domain score (rho = 0.364). There was no significant correlation between the SA-PR and FSFI Satisfaction subscale scores. The SA-CS subscale score was significantly correlated with only the FSFI Satisfaction and Pain domain scores (rho = 0.209 and 0.278, respectively). Higher quality of sexual function on PISQ-IR was correlated with better FSFI scores.

An analysis of the questionnaires evaluating general QoL demonstrated that selected SF-36 domain scores were positively correlated with SA-CS, SA-GQ and SA-CI scores. Less disability in the QoL assessment was correlated with better sexual function on PISQ-IR. Similar correlations were found for KHQ scores. A statistically significant negative correlation was found between selected KHQ domain scores and SA-AO, SA-CS, SA-GQ and SA-CI scores. Lower KHQ scores (better health status) corresponded with higher SA PISQ-IR scores (better sexual function). Detailed results are presented in Table 5.

**Table 5:** Criterion validity: scale score correlations in SA women

	Arousal, orgasm	Partner related	Condition specific	Global quality	Condition impact	Desire	Summary score
POP-Q stage	0.004	−0.097	0.138	0.028	0.026	−0.087	0.039
Pelvic floor tone	−0.067	−0.022	−0.080	−0.183*	−0.114	−0.028	−0.118
Oxford scale	0.099	0.111	−0.014	0.118	0.034	−0.029	0.086
Incontinence Severity Index	−0.026	0.051	−0.361**	−0.096	−0.225*	−0.026	−0.160
PFDI-20	−0.333**	−0.343**	−0.284**	−0.357**	−0.367**	−0.141	−0.403**
EPIC question 35^a^	0.001	−0.034	0.012	−0.028	−0.197	0.260*	0.003
Body mass index (kg/m^2^)	−0.166	−0.031	−0.054	−0.064	−0.113	−0.029	−0.156
SF-36 scales							
Physical functioning	0.210*	0.007	0.218*	0.152	0.427**	−0.020	0.253*
Role-physical	0.144	−0.048	0.076	0.208*	0.384**	−0.010	0.217*
Role-emotional	0.149	−0.026	0.249*	0.313**	0.440**	0.066	0.325**
Vitality	0.148	0.050	0.235*	0.378**	0.422**	0.047	0.340**
Mental health	0.162	0.015	0.328**	0.287**	0.531**	0.037	0.355**
Social functioning	0.138	0.026	0.263*	0.344**	0.405**	−0.028	0.289**
Bodily pain	0.242*	0.129	0.124	0.328**	0.443**	0.061	0.327**
General health	0.133	−0.028	0.149	0.215*	0.383**	0.115	0.249*
KHQ^b^							
General health perception	−0.248*	−0.107	−0.151	−0.301**	−0.326**	−0.117	−0.245**
Incontinence impact	−0.175	0.058	−0.401**	−0.272*	−0.483**	0.122	−0.273**
Role limitations	−0.229*	0.029	−0.422**	−0.287*	−0.514**	0.075	−0.318**
Physical limitations	−0.193	0.010	−0.388**	−0.271*	−0.505**	0.062	−0.304**
Social limitations	−0.177	0.018	−0.303**	−0.190	−0.436**	0.088	−0.307**
Personal limitations	−0.382**	−0.127	−0.374**	−0.376**	−0.624**	−0.079	−0.458**
Emotional problems	−0.230*	−0.005	−0.471**	−0.308**	−0.532**	0.047	−0.350**
Sleep/energy	−0.307**	−0.167	−0.138	−0.229*	−0.370**	0.023	−0.254**
Symptom severity	−0.197	0.018	−0.345**	−0.243*	−0.481**	0.035	−0.295**
FSFI							
Desire	0.570**	0.173	0.057	0.344**	0.253**	0.705**	0.496**
Arousal	0.684**	0.364**	0.011	0.502**	0.317**	0.638**	0.611**
Lubrication	0.464**	0.136	−0.086	0.260**	0.194*	0.370**	0.344**
Orgasm	0.713**	0.174	0.147	0.458**	0.352**	0.496**	0.580**
Satisfaction	0.586**	0.156	0.209*	0.572**	0.407**	0.363**	0.613**
Pain	0.475**	0.102	0.278**	0.401**	0.434**	0.223*	0.499**
Total score	0.736**	0.229*	0.115	0.522**	0.389**	0.573**	0.646**

*POP* pelvic organ prolapse, *POP-Q* Pelvic Organ Prolapse Qualification, *PFDI-20* Pelvic Floor Distress Inventory, *EPIC* Epidemiology of Prolapse and Incontinence Questionnaire, *KHQ* King’s Health Questionnaire, *SF-36* a 36-item Short Form Health Survey, *FSFI* Female Sexual Function Index

*p < 0.05, **p < 0.01

^a^Analysis only in patients with POP

^b^Assessed only in patients with UI

The summary SA score was significantly correlated with all domain scores in the following questionnaires: SF-36, KHQ and FSFI (including FSFI total score). The summary SA score was not correlated with physical examination measures, but was negatively correlated with PFDI-20 scores.

## Discussion

According to IUGA, the PISQ-IR is currently being validated in numerous countries. Thus, it seems reasonable to hope that the questionnaire will soon become a widely popular tool, allowing comparison of study results in different populations. However, at present, only a few language versions other than English are available (Mandarin Chinese, Arabic, German, Hungarian, Spanish and Czech) [16–22].

Internal consistency measured with Cronbach’s *α* assesses how closely related a set of items is as a group. The resulting *α* coefficient of reliability ranges from 0 (the scale items are entirely independent) to 1 (all of the items have high covariances). A score of >0.7 indicates adequate scale reliability. The PISQ-IR Polish version proved to have strong internal consistency in NSA and SA women. The Cronbach’s *α* indicated moderate internal consistency only in two subscales, NSA-CS and SA-PR (*α* = 0.651 and 0.605, respectively). In the original English language version of PISQ-IR, Cronbach’s *α* values of <0.7 were found for SA-CS (*α* = 0.63) [3]. The test–retest analysis of the PISQ-IR Polish version revealed a strong correlation between the paired test–retest answers. The English version of the questionnaire, as well as other language versions, also shows stability over time, with no items demonstrating differences between test and retest [3, 16].

According to the scale scoring when reporting PISQ-IR results, six and four separate scale scores are reported for SA and NSA women, respectively. There are two recommended methods for presenting results: a mean calculation or a transformed sum score [23]. However, some authors have chosen analysis of summary PISQ-IR scores, which no doubt facilitates comparative analysis [20]. In a recently published study, a single summary score for use in SA women was introduced [24], and is the reason a single summary SA score was also used in our study. Furthermore, the obtained results confirmed a correlation between the single summary SA score and the scores from the other questionnaires used in this study.

Criterion-related validity was based on the analysis of correlations between PISQ-IR scores and clinical measures. In our study, in both groups (SA and NSA) neither the -CS scores nor the -CI scores (the two condition-specific subscales) were correlated with clinical measures. Similar results have been reported for the Spanish and Arabic versions of the questionnaire [20, 21]. In the original version, the questionnaire scores for NSA women did not correlate with clinical measures, whereas a weak correlation was found for SA women between POP-Q stage and SA-CS and SA-CI scores [3]. Various authors have reported no association between sexual function and data from the physical examination, i.e. POP stage, but found a link between the perception of body image and distress level caused by a given condition [25, 26].

Concerning the self-reported condition-specific measures (PFDI-20, ISI, EPIQ question #35) for criterion validity in NSA women, there were moderately significant correlations between PFDI-20 scores and NSA-CS and NSA-CI scores and between EPIC question #35 score with NSA-CI score. There were no correlations between ISI and condition-specific subscale scores in NSA women, while in SA women there were significant negative correlations between condition-specific subscale scores (i.e. SA-CS, SA-CI) and PFDI-20 scores and ISI. The observed correlations were weak or moderate, which is consistent with the results found for the original version of the questionnaire and other language versions of PISQ-IR, for which the results did not always reach statistical significance [16, 18–20]. The Arabic version, with a strong correlation between ISI and NSA-CI and SA-CS scores, is the only exception so far, probably due to a high proportion of women with UI in the investigated population [21].

The general questionnaire which evaluates sexual function, i.e. FSFI, was used for criterion validity among SA women. All PISQ-IR SA subscale scores, except the SA-CS score, were significantly correlated with the FSFI total score. Analysis of correlations between the same domains of the two questionnaires as presented by Rogers et al. [3], and comparison of similar aspects of sexual life, revealed that the Polish version of PISQ-IR generated statistically significant results similar to those of the original version. SA-AO scores were correlated with FSFI Desire, Arousal, Lubrication, Orgasm and Pain domain scores. SA-CS scores were correlated with FSFI Satisfaction and Pain domain scores. SA-GQ scores were correlated with FSFI Desire, Arousal and Satisfaction scores. SA-CI scores were correlated with FSFI Arousal and Pain scores. SA-D scores were correlated with the FSFI Desire domain scores. However, unlike the original version, SA-PR subscale scores were not correlated with the FSFI Satisfaction domain scores in the Polish version.

Health-related QoL questionnaires, i.e. SF-36 (assessing general health) and KHQ (for patients with UI), confirmed that less disability in QoL assessment was correlated with better sexual function in PISQ-IR in SA women. Interestingly, KHQ General heath perception scores were correlated negatively with SA-AO, SA-GQ and SA-CI scores, while no such correlations were observed among NSA women. SA women with lower quality of sexual function also rated their general health as ‘worse’. The utility of the PISQ-IR single summary score for SA was confirmed: it correlated with all subscales of the questionnaires applied (FSFI, SF-36, KHQ, PFDI-20). Cronbach’s *α* was also very high (*α* = 0.9), which is similar to the value found by Mestre et al. (*α* = 0.91) [20].

Questions #4 and #19 turned out to be problematic in the process of translation and validation in Polish. In these questions, the patients self-assess their sexual life on a scale from 1 to 5, with ‘1’ for ‘satisfied about sex life’ and ‘5’ for ‘dissatisfied’. The respondents found this presentation to be very misleading as it is the reverse of the school grades (‘1’ for failed and ‘5’ for excellent) and, as a result, the answers were also reversed. This problem was remedied by a different graphical representation. German authors have also encountered problems with these questions. They found that some respondents did not answer these questions so they modified the answering formats to unify them with the remaining questions [18].

PISQ-IR has no imposed time limit and it is the respondent who decides whether or not she considers herself ‘sexually active’, while for the FSFI, which was used as criterion validity, the questions pertain to the last four weeks, which might have been the source of discrepancies in self-assessed sexual activity. This resulted in some ambiguity and confusion during the process of completion and the matter had to be clarified. As in the study group, premenopausal women were instructed not to have sexual intercourse for one month prior to surgery. In daily clinical practice, it seems advisable to leave the self-assessment of sexual activity to the patient rather than to impose the time criterion. In FSFI scoring, even a single answer of ‘0’ is enough to include a patient in the NSA group, whereas according to PISQ-IR a respondent can be regarded as SA even if, for example, she has no partner, thus allowing a broader perspective on sexual life.

High response rate (82.1%) and meeting the expected sample size in both, SA and NSA populations are definite strengths of our study. Most probably it is the result of a direct contact with the doctor and detailed presentation of the aims of the study, which was conducted in a university-based clinic, where academic activity of the doctors is expected. The validation process presents an opportunity to talk to the patient in detail and thoroughly analyze the problems associated with her PFD. Often, the women mentioned that completion of the questionnaires made them realize the actual effect their disease had on their health-related QoL and proper timing of surgical treatment.

### Conclusions

PISQ-IR Polish version is a valid, stable and reliable tool for assessing sexual function in SA and NSA women with PFD and can be used in practice.

## Electronic supplementary material

ESM 1 (PDF 427 kb)
