# Supplementary material for: Polish translation and validation of the Pelvic Organ Prolapse/Urinary Incontinence Sexual Questionnaire, IUGA-Revised (PISQ-IR)
Source: Int Urogynecol J. 2017 Dec 29;30(1):55–64. doi: 10.1007/s00192-017-3539-5 (PMC6761085; doi:10.1007/s00192-017-3539-5)
Supplement: Supplementary file 1 — (PDF 427 kb) [file 192_2017_3539_MOESM1_ESM.pdf]

Kwestionariusz PISQ-IR:
Seksualne Funkcjonowanie Kobiet z Wypadaniem Narządów Miednicy Mniejszej, Nietrzymaniem Moczu i/lub Nietrzymaniem Stolca

Inicjały : __ __

Data urodzenia: __ / __ / ____

Data wypełnienia kwestionariusza: __ / __ / ____

Pytanie 1. Które z poniższych najlepiej Panią opisuje:

całkowicie nieaktywna seksualnie **1** □ → Proszę przejść do Pytania 2 – **Sekcja 1: Brak Aktywności Seksualnej**

aktywna seksualnie z lub bez partnera **2** □→ Proszę przejść do Pytania 7 – **Sekcja 2: Aktywność Seksualna**

SEKCJA 1: BRAK AKTYWNOŚCI SEKSUALNEJ

Jeśli jest Pani aktywna seksualnie proszę zaznaczyć tę rubrykę □ i przejść do **Sekcji 2** na stronie 3.

Pytanie 2. Poniżej znajduje się lista powodów, dla których może Pani być nieaktywna seksualnie. Proszę przy każdym zaznaczyć do jakiego stopnia się Pani zgadza lub nie zgadza z opinią, że to jest powód Pani braku aktywności seksualnej.

		**Zdecydowanie się zgadzam**	**Raczej się zgadzam**	**Raczej się nie zgadzam**	**Zdecydowanie się nie zgadzam**
a.	Brak partnera	□ 1	□ 2	□ 3	□ 4
b.	Brak zainteresowania	□ 1	□ 2	□ 3	□ 4
c.	Z powodu problemów z pęcherzem moczowym i jelitami (nietrzymanie moczu lub stolca) lub z powodu obniżenia narządów płciowych (uczucie „wybrzuszenia” w pochwie)	□ 1	□ 2	□ 3	□ 4
d.	Inne moje problemy zdrowotne	□ 1	□ 2	□ 3	□ 4
e.	Ból	□ 1	□ 2	□ 3	□ 4

Pytanie 3. Do jakiego stopnia **obawa** przed nietrzymaniem moczu i/lub stolca i/lub uczuciem „wybrzuszenia” w pochwie (obniżenia pęcherza moczowego, odbytnicy, wypadania macicy) powodują, że unika Pani lub ogranicza swoją aktywność seksualną?

1 □ wcale
2 □ trochę
3 □ znacznie
4 □ bardzo

Pytanie 4. Dla każdego z poniższych, proszę zaznaczyć krzyżykiem na osi liczbę, która najlepiej pokazuje jaką ma Pani opinię na temat swojego życia seksualnego.

a. Zadowolona ◄—1—2—3—4—5—► Niezadowolona

b. Wystarczające ◄—1—2—3—4—5—► Niewystarczające

Pytanie 5. Do jakiego stopnia się Pani zgadza lub nie zgadza z każdą z poniższych opinii:

		**Zdecydowanie się zgadzam**	**Raczej się zgadzam**	**Raczej się nie zgadzam**	**Zdecydowanie się nie zgadzam**
a.	Czuję się sfrustrowana moim życiem seksualnym	☐ 1	☐ 2	☐ 3	☐ 4
b.	Czuję się seksualnie gorsza z powodu moich problemów z nietrzymaniem i/lub obniżeniem narządów płciowych	☐ 1	☐ 2	☐ 3	☐ 4
c.	Czuję się rozgniewana z powodu wpływu, jaki nietrzymanie i/lub obniżenie narządów płciowych ma na moje życie seksualne	☐ 1	☐ 2	☐ 3	☐ 4

Pytanie 6. Jak bardzo przeszkadza Pani brak aktywności seksualnej?

1 ☐ wcale
2 ☐ trochę
3 ☐ znacznie
4 ☐ bardzo

Koniec pytań dla nieaktywnych seksualnie

SEKCJA 2: AKTYWNOŚĆ SEKSUALNA
Pozostałe pytania w kwestionariuszu dotyczą tematów rzadko poruszanych w ankietach.
Pani odpowiedzi pozostaną poufne. Proszę odpowiedzieć na nie tak rzetelnie i szczerze jak tylko Pani może.

Pytanie 7. Jak często odczuwa Pani pobudzenie seksualne (fizyczne lub psychiczne podniecenie) podczas aktywności seksualnej?

1 □ nigdy
2 □ rzadko
3 □ czasami
4 □ zazwyczaj
5 □ zawsze

Pytanie 8. Podczas angażowania się w czynności seksualne, jak często odczuwa Pani poniższe:

	**nigdy**	**rzadko**	**czasami**	**zazwyczaj**	**prawie zawsze**
a. Spełnienie	□ 1	□ 2	□ 3	□ 4	□ 5
b. Zawstydzenie	□ 1	□ 2	□ 3	□ 4	□ 5
c. Strach	□ 1	□ 2	□ 3	□ 4	□ 5

Pytanie 9. Jak często zdarza się Pani popuścić mocz i/lub stolec podczas wykonywania jakichkolwiek czynności seksualnych?

1 □ nigdy
2 □ rzadko
3 □ czasami
4 □ zazwyczaj
5 □ zawsze

Pytanie 10. W porównaniu do orgazmów które miała Pani w przeszłości, jak intensywne są Pani obecne orgazmy?

1 □ zdecydowanie mniej intensywne
2 □ mniej intensywne
3 □ taka sama intensywność
4 □ bardziej intensywne
5 □ zdecydowanie bardziej intensywne

Pytanie 11. Jak często odczuwa Pani ból podczas stosunku płciowego? (Jeśli nie miewa Pani stosunków, proszę zaznaczyć tę rubrykę ☐ i przejść do następnego pytania).

1 ☐ nigdy
2 ☐ rzadko
3 ☐ czasami
4 ☐ zazwyczaj
5 ☐ zawsze

Pytanie 12. Czy ma Pani partnera seksualnego?

1 ☐ TAK proszę przejść do Pytania 13
2 ☐ NIE proszę przejść do Pytania 15

Pytanie 13. Jak często Pani partner ma problemy (brak podniecenia, pożądania, erekcji, etc.), które ograniczają Pani aktywność seksualną?

1 ☐ cały czas
2 ☐ przez większość czasu
3 ☐ czasami
4 ☐ prawie nigdy/rzadko

Pytanie 14. Czy w Pani ogólnym odczuciu, Pani partner ma pozytywny czy negatywny wpływ na każde z poniższych:

	**Bardzo pozytywny**	**Raczej pozytywny**	**Raczej negatywny**	**Bardzo negatywny**
a. Pani pożądanie seksualne	☐ 1	☐ 2	☐ 3	☐ 4
b. Częstotliwość Pani aktywności seksualnej	☐ 1	☐ 2	☐ 3	☐ 4

Pytanie 15. Kiedy jest Pani w trakcie wykonywania czynności seksualnych, jak często czuje Pani, że chciałaby więcej?

1 ☐ nigdy
2 ☐ rzadko
3 ☐ czasami
4 ☐ zazwyczaj
5 ☐ zawsze

Pytanie 16. Jak często odczuwa Pani pożądanie seksualne, włączając w to ochotę na uprawianie seksu, myśli i fantazje seksualne, etc.?
1 ☐ codziennie
2 ☐ raz w tygodniu
3 ☐ raz w miesiącu
4 ☐ rzadziej niż raz w miesiącu
5 ☐ nigdy

Pytanie 17. Jak oceniłaby Pani swój poziom (stopień) pożądania lub zainteresowania seksualnego?

1 □ bardzo wysoki
2 □ wysoki
3 □ umiarkowany
4 □ niski
5 □ bardzo niski lub zerowy

Pytanie 18. Do jakiego stopnia obawa przed nietrzymaniem moczu, stolca i/lub „wybrzuszeniem” w pochwie (obniżenie narządów płciowych) powoduje, że unika Pani aktywności seksualnej?

1 □ wcale
2 □ trochę
3 □ znacznie
4 □ bardzo

Pytanie 19. Dla każdego z poniższych, proszę <u>zaznaczyć krzyżykiem na osi liczbę</u>, która najlepiej pokazuje jaką ma Pani **opinię na temat swojego życia seksualnego**.

a.	Zadowolona	1	2	3	4	5	Niezadowolona
b.	Wystarczające	1	2	3	4	5	Niewystarczające
c.	Pewna siebie	1	2	3	4	5	Niepewna siebie

Pytanie 20. Do jakiego stopnia się Pani zgadza lub nie zgadza z każdą z poniższych opinii:

		**Zdecydowanie się zgadzam**	**Raczej się zgadzam**	**Raczej się <u>nie</u> zgadzam**	**Zdecydowanie się <u>nie</u> zgadzam**
a.	Czuję się sfrustrowana moim życiem seksualnym	□ 1	□ 2	□ 3	□ 4
b.	Czuję się seksualnie gorsza z powodu moich problemów z nietrzymaniem i/lub obniżeniem narządów płciowych	□ 1	□ 2	□ 3	□ 4
c.	Czuję się zażenowana moim życiem seksualnym	□ 1	□ 2	□ 3	□ 4
d.	Czuję się rozgniewana z powodu wpływu, jaki nietrzymanie i/lub obniżenie narządów płciowych ma na moje życie seksualne	□ 1	□ 2	□ 3	□ 4
